# Telemedicine for HIV care: a cross-sectional survey of people living with HIV receiving care at two federally qualified health centers in Los Angeles during a mature phase of the COVID-19 pandemic

**DOI:** 10.1186/s12879-024-10351-x

**Published:** 2024-12-31

**Authors:** Daisy Walker, Corrina Moucheraud, Derrick Butler, Christian Takayama, Steven Shoptaw, Judith S. Currier, Jay Gladstein, Risa Hoffman

**Affiliations:** 1 Division of Infectious Diseases, David Geffen School of Medicine at UCLA, Los Angeles, CA US; 2 School of Global Public Health, New York University, New York, NY US; 3 To Help Everyone, Los Angeles, CA US; 4 APLA Health, Los Angeles, CA US; 5 Department of Family Medicine, David Geffen School of Medicine at UCLA, Los Angeles, CA US

**Keywords:** HIV, Telemedicine, Telehealth, Los Angeles

## Abstract

**Background:**

The COVID-19 pandemic resulted in the rapid implementation of telemedicine for HIV care at federally qualified health centers (FQHCs) in the United States. We sought to understand use of telemedicine (telephone and video) at two FQHCs in Los Angeles, and the client attitudes towards and experiences with telemedicine as part of future HIV care.

**Methods:**

We conducted surveys with 271 people living with HIV (PLHIV), with questions covering sociodemographic factors, telemedicine attitudes and experiences, technological literacy, and access to technological resources and privacy. Survey data were analyzed utilizing summary statistics, chi-square analyses, and Fisher’s exact test to understand associations between sociodemographic factors and telemedicine attitudes and experiences.

**Results:**

Sixty percent of the sample had used any telemedicine and, of these, 93% utilized only telephone visits. Almost all respondents (95%, *n* = 257) had access to a functioning smartphone and self-rated their technological literacy as high. Most had consistent access to privacy (88%, *n* = 239), and those without privacy noted this as a barrier to the use of telemedicine. The main benefits of telemedicine (compared to in person) were savings of time and money, convenience, and ability to complete appointments as scheduled. Just over half of PLHIV said they would feel more comfortable discussing sensitive topics (e.g., substance use, relationship issues) in person than over telephone (60%, *n* = 164) or video (55%, *n* = 151). Despite limited experience with video telemedicine, half of all participants desired a mix of telephone and video visits as part of their future HIV care.

**Conclusions:**

During a mature phase of the COVID-19 pandemic, PLHIV in our study showed high satisfaction with telemedicine, largely conducted as telephone visits, and high interest in telemedicine visits as a component of their future HIV care. Future studies should explore barriers to implementing video telemedicine in FQHCs and determine telemedicine’s impact on clinical outcomes, including engagement and viral suppression.

**Supplementary Information:**

The online version contains supplementary material available at 10.1186/s12879-024-10351-x.

## Background

The arrival of the COVID-19 pandemic marked the beginning of telemedicine use for many people living with HIV (PLHIV) who previously had received care exclusively in person [1]. While many private practice and academic settings had provided some degree of telemedicine pre-pandemic, most federally qualified health centers (FQHCs) offered telemedicine for the first time during the pandemic, due to regulatory waivers provided by the Centers for Medicare & Medicaid Services (CMS), which allowed these clinics to receive compensation for visits conducted via telemedicine, whether telephone (audio-only) or video [2]. In the United States, stay-at-home orders beginning in early 2020 made telemedicine the only way that some PLHIV could be in contact with their clinicians [3].

While telemedicine has been shown to be an effective mode of care for several chronic conditions in various care settings (FQHCs, veterans affairs, academic, correctional, etc.) resulting in clinical and cost outcomes that are equal to, or better than, in-person care [4–9], telemedicine has not been as widely studied for HIV care, especially HIV care within FQHCs. These clinics serve people who face many barriers to care and have been historically marginalized within the health care system and broader society [10].

Reports of telemedicine at FQHCs to date, including those caring for PLHIV, have shown a disproportionately high volume of telephone visits relative to video visits [3, 11]. Telephone visits may be associated with poorer quality care relative to video, and lack of access to video telemedicine may widen health disparities [12]; however, telephone visits have become popular in the COVID-19 era because they are easily implemented by clinicians and have lower barriers for clients. It is thus possible that requiring video telemedicine, particularly in FQHCs, could increase health inequities [13].

We sought to understand telemedicine use and PLHIV perspectives about telemedicine at two FQHCs in Los Angeles during a mature phase of the COVID-19 pandemic when telemedicine was offered routinely to this population.

## Methods

### Definitions

We define “telephone telemedicine” as a health care visit between a clinician and patient that occurs via a phone call, where only audio (no video) is used. “Video telemedicine” is defined as a health care visit that occurs via a video call, where the provider and patient are visible to each other during the clinical encounter. The term “telemedicine” is used broadly to encompass both telephone and video telemedicine.

### Setting and population

From March to November 2022, we performed a cross-sectional survey with 271 PLHIV at two FQHCs in south Los Angeles County’s Service Planning Area (SPA) 6. The rate of new HIV infections in SPA 6 is the second highest in the county, with a below-average viral suppression rate of 59% [14]. The population cared for in these two FQHCs experiences high rates of unemployment, housing instability, substance use, and mental health disorders [10]. Both clinics offered telemedicine visits starting in March 2020 with the beginning of the COVID-19 stay-at-home orders and continued to offer either telephone or video telemedicine (at the clinicians’ discretion) through the survey period.

To be eligible for the survey, participants had to be at least 18 years old, and receiving HIV care at one of the two study clinics for at least 3 months. Individuals were recruited during routine in-person and telemedicine clinic encounters by HIV clinicians or case managers, and those who were interested were referred to the study coordinator for screening.

### Conceptual framework and survey development

The conceptual framework was derived from two existing models: Venkatesh et al., which was developed to assess “the drivers of acceptance in order to proactively design interventions targeted at populations of users that may be less inclined to adopt and use new systems [15]”; and the Theory of Planned Behavior [16].

Survey domains were aligned with our conceptual framework and included sociodemographic characteristics; costs of seeking routine HIV care (transportation, opportunity costs); questions about access to technological resources (including cost of maintaining telephone and Wi-Fi plans) and privacy for telemedicine visits; previous offer and use of telemedicine including mode (telephone versus video); and questions about attitudes towards and experiences with telemedicine. We also asked participants to rate their technological literacy [17, 18], using a scale of 1–10 with 1 being “not at all able to” and 10 being “totally able to” for several devices (telephone, tablet, laptop, and/or desktop computer). Finally, participants were asked about their preferences for future telemedicine use. The survey tool is included in Supplementary Table 1.

### Data collection and analysis

Surveys were conducted in person or by telephone (based on participant preference) and lasted no more than one hour. Surveys were done in either English or Spanish by one research team member (DW). Participants received $40 cash as compensation for completing the survey. Those that completed the survey in-person were given $40 cash following survey completion, and those that completed the survey by telephone came to the clinic at their earliest convenience or at their next appointment to pick up their $40 cash from a member of the study team or a designated proxy at the clinic.

We used summary statistics to describe participant demographic data and survey responses. Based on our conceptual framework, we hypothesized that clients with less education, unstable housing, lower technological literacy, those with Spanish as their primary language, and older individuals would be more likely to experience barriers to telemedicine and have less favorable attitudes about telemedicine, particularly video telemedicine. We evaluated associations of demographics (age, gender, education level, preferred language, and housing status) with telemedicine attitudes and experiences using chi square and Fisher’s exact tests. For housing status, we defined stable housing as owning, renting, or sharing (but not paying for) housing. To measure attitudes about and experiences with telemedicine, participants were given several statements concerning telemedicine (e.g. “Telemedicine (is/can be) more convenient than in-person visits”) and were asked to rate each statement on the following Likert scale: Strongly disagree, Somewhat disagree, Neutral, Somewhat agree, or Strongly Agree. The statements utilized are shown in Supplementary Table 1. Viral suppression was defined as < 20 copies/mL. All analyses use the full sample; we also performed a sensitivity analysis that included only those participants who reported actual telemedicine experience. The study was approved by the Institutional Review Board at the University of California, Los Angeles (IRB #20-001508).

## Results

### Demographics

The median age of respondents was 49 years (IQR 37, 58), and 79% (*n* = 215) identified as cisgender men, 17% (*n* = 47) as cisgender women, 2% (*n* = 6) as transgender women and 1% (*n* = 3) as gender nonconforming individuals. Most PLHIV identified as either Black/African American (46%, *n* = 126) or as Hispanic or Latino/a (26%, *n* = 71). The majority of participants reported English as their preferred language (85%, *n* = 232) with the remainder reporting either Spanish (14%, *n* = 37) or another language (1%, *n* = 2). The majority of individuals had at minimum completed high school or received their GED (79%, *n* = 216), and 52% (*n* = 141) were unemployed or on disability, while 48% (*n* = 130) were employed or retired. Most respondents (85%, *n* = 230) reported having stable housing, but one-third (33%, *n* = 80) were worried about maintaining their housing in the next three months. Participants were living with HIV for a median of 12 years (IQR 6, 21) and were on antiretroviral therapy (ART) for a median of 10 years (IQR 5, 19). Table 1 summarizes demographic and clinical data.

**Table 1:** Sociodemographic and clinical characteristics of PLHIV (*N* = 271)

	Overall	Clinic 1	Clinic 2
	(*N* = 271)	(*N* = 136)	(*N* = 135)
**Age**, Median (IQR)	49 (36–58)	44 (33.5–56)	52 (39–61)
**Gender**, *N* (%)			
Cisgender female	47 (17)	13 (10)	34 (25)
Cisgender male	215 (79)	115 (84)	100 (74)
Transgender female	6 (2)	5 (4)	1 (1)
Gender nonconforming	3 (1)	3 (2)	0 (0)
**Race/Ethnicity**, *N* (%)			
Black or African American	126 (46)	57 (42)	69 (51)
Hispanic or Latino/a	71 (26)	25 (18)	46 (34)
Asian	6 (2)	5 (4)	1 (1)
Native American	3 (1)	2 (1)	1 (1)
White/Caucasian	27 (10)	21 (15)	6 (4)
Multi-racial	35 (13)	23 (17)	12 (9)
Other	2 (1)	2 (1)	0 (0)
Prefer not to answer	1 (1)	1 (1)	0 (0)
**Preferred language**, *N* (%)			
English	232 (85)	126 (93)	106 (79)
Spanish	37 (14)	9 (6)	28 (20)
Other	2 (1)	1 (1)	1 (1)
**Sexual orientation**, *N* (%)			
Heterosexual	96 (35)	33 (24)	63 (46)
Gay	121 (45)	70 (51)	51 (38)
Bisexual	31 (11)	19 (14)	12 (9)
Other (queer, pansexual, asexual)	12 (4)	8 (6)	4 (3)
Prefer not to answer	11 (4)	6 (4)	5 (4)
**Highest level of school completed**, *N* (%)			
Some school but did not complete high school or GED	55 (20)	19 (14)	36 (27)
High school or GED	149 (55)	78 (57)	71 (52)
College or university	58 (21)	31 (23)	27 (20)
Graduate studies	9 (3)	8 (6)	1 (1)
**Employment status**, *N* (%)			
Working - part-time	37 (14)	15 (11)	22 (16)
Working - full-time	75 (28)	45 (33)	30 (22)
Not working - retired	18 (6)	10 (7)	8 (6)
Not working - on disability	56 (21)	25 (18)	31 (23)
Not working - unemployed	85 (31)	41 (30)	44 (33)
**Housing status**, *N* (%)			
Stable*	230 (85)	112 (82)	118 (88)
Unstable**	40 (14)	23 (16)	17 (12)
Prefer not to answer	1 (1)	1 (1)	0 (0)
**If stable housing**,*** worried about maintaining housing in next three months, *N* (%)			
Yes, very worried	47 (19)	22 (19)	25 (20)
Somewhat worried	33 (14)	17 (14)	16 (13)
No, not worried at all	158 (66)	79 (67)	79 (65)
Prefer not to answer	2 (1)	0 (0)	2 (2)
**Time living with HIV (years)**, Median (IQR)	12 (6–21)	10 (5-19.5)	14 (6–22)
**Time on ART (years)**, Median (IQR)	10 (5–19)	8 (4.5–18)	13 (6–22)

*Stable housing is defined as owning, renting, or sharing but not paying for housing; **Unstable housing is defined as staying in transitional housing, a shelter, in a car, or on the street; ***N = 230

Of those who had completed bloodwork in the 12 months prior to the survey (97%, *n* = 264), 69% (*n* = 182) were virally suppressed (< 20 copies/mL), 21% (*n* = 55) had a viral load between 20 and 1000 copies/mL, and 10% (*n* = 27) had a viral load greater than 1000 copies/mL. The median CD4 count was 583 (IQR 396, 803).

60% (*n* = 162) of PLHIV had used telemedicine for HIV care, which was predominantly done by telephone only (56%, *n* = 151), with only 4% (*n* = 11) reporting a video visit (Table 2). Of those who had used telephone visits for their HIV care, 95% (*n* = 152) felt satisfied with the quality of care of these visits, while all respondents reporting a video visit felt satisfied with the quality of care. Most individuals (61%, *n* = 166) had no experience using telemedicine for non-HIV-related types of care, but those who did have this experience reported using it the most for mental health care (46%, *n* = 48).

**Table 2:** Telemedicine use for HIV and non-HIV care (*N* = 271)

	Overall	Clinic 1	Clinic 2
	(*N* = 271)	(*N* = 136)	(*N* = 135)
**Experience with telemedicine for HIV care (ever)**, *N* (%)			
Yes, telephone only	151 (56)	76 (56)	75 (56)
Yes, video only	2 (1)	0 (0)	2 (1)
Yes, both telephone and video	9 (3)	2 (1)	7 (5)
No	109 (40)	58 (43)	51 (38)
**Experience with telemedicine for non-HIV care (in past year)**, *N* (%)			
Yes, telephone only	52 (19)	27 (20)	25 (18)
Yes, video only	34 (13)	23 (17)	11 (8)
Yes, both telephone and video	19 (7)	14 (10)	5 (4)
No	166 (61)	72 (53)	94 (70)
**If ever used non-HIV care via telemedicine**,* types of care, *N* (%)			
Mental health care	48 (46)	30 (47)	18 (44)
Case management	13 (12)	7 (11)	6 (15)
Acute care (sinus infection, cold, flu, etc.)	4 (4)	1 (2)	3 (7)
COVID-related care	4 (4)	4 (6)	0 (0)
Specialty care (cardiology, gynecology, nephrology, etc.)	16 (15)	6 (9)	10 (24)
Multiple types of care	20 (19)	16 (25)	4 (10)

*N = 105

**Table 3:** Opportunity costs and cost of technological resources (*N* = 271)

	Overall	Clinic 1	Clinic 2
	(*N* = 271)	(*N* = 136)	(*N* = 135)
**Takes time off from work to attend visits**, *N* (%)			
Yes, paid time off	8 (3)	6 (4)	2 (1)
Yes, unpaid time off	38 (14)	19 (14)	19 (14)
Sometimes, paid time off	2 (1)	1 (1)	1 (1)
Sometimes, unpaid time off	9 (3)	5 (4)	4 (3)
Sometimes, varies between paid and unpaid time off	2 (1)	0 (0)	2 (1)
No	211 (77)	104 (76)	107 (79)
Prefer not to answer	1 (1)	1 (1)	0 (0)
**Time spent at clinic for a visit (minutes)**, Median (IQR)	60 (45–120)	45 (30–60)	90 (60–120)
**Lost wage for time off from work for one visit (dollars)**,* Median (IQR)	60 (40–100)	75 (50–120)	50 (35–100)
**Pays for phone and/or data**, *N* (%)			
Yes, monthly plan	205 (77)	103 (79)	102 (76)
Yes, pay as you go	2 (1)	0	2 (1)
Yes, other	1 (1)	1 (1)	0 (0)
No	58 (21)	27 (20)	31 (23)
**Total cost of phone each month [data + device] (dollars)**,** Median (IQR)	60 (50–104)	70 (51-115.5)	57 (47.5–89)
How difficult is it to financially maintain device? *** Median (IQR)	3 (1–7)	3 (1–7)	2 (1-7.5)
How difficult is it to financially maintain data? *** Median (IQR)	3 (1–7)	2 (1–7)	5 (1–8)
**Pays for Wi-Fi**, *N* (%)			
Yes, separate from phone plan	112 (41)	62 (46)	50 (37)
Yes, bundled with phone plan	38 (14)	18 (13)	20 (15)
No	121 (45)	56 (41)	65 (48)
**Total cost of Wi-Fi each month (dollars)**, Median (IQR)	54 (35–80)	55 (37.5–80)	52 (30–79)
How difficult is it to financially maintain Wi-Fi? **** Median (IQR)	5 (1–8)	5 (1–7)	5 (1–8)

*N = 49; **N = 208; ***N = 208, measured on a scale from 1 to 10 (with 1 being Not at all difficult and 10 being Extremely difficult); ****N = 150, measured on a scale from 1 to 10 (with 1 being Not at all difficult and 10 being Extremely difficult)

Approximately half (46%, *n* = 126) of participants traveled to the clinic in a personal vehicle, with the remainder using either public transportation (23%, *n* = 62) or ridesharing apps (15%, *n* = 40). The median time spent traveling to the clinic from home was 30 min (IQR 15, 45) and the median cost per one-way trip among those who paid (69%, *n* = 189) was $5 (IQR $3, $10).

### Access to telemedicine

Almost all PLHIV currently owned a smartphone (95%, *n* = 257) and had consistent access to a telephone over the past three months (95%, *n* = 256). Almost all participants (97%, *n* = 264) reported that they had access to reliable cellular data or Wi-Fi. Just over half of participants (58%, *n* = 158) reported that they currently owned either a tablet or a laptop or a desktop computer. Of the remaining respondents who did not own a tablet, laptop or computer (42%, *n* = 113), 53% (*n* = 60) said they would never be able to borrow one, 27% (*n* = 31) would be able to borrow one sometimes, and 19% (*n* = 22) always.

The majority of PLHIV interviewed were the primary payor of their telephone and/or data plans (79%, *n* = 208) and Wi-Fi plan (55%, *n* = 150) (Table 3). The median total cost of telephone each month (including both device and data costs) was $60 (IQR $50, $100) and for Wi-Fi was $54 (IQR $35, $80) per month. Overall, individuals found it slightly easier to financially maintain their telephone/data plan compared to their Wi-Fi.

Most PLHIV had access to privacy for telephone visits for their HIV care always (88%, *n* = 239). Of the 32 people who did not always have access, 31% (*n* = 10) reported that this was always, and 28% (*n* = 9) reported it was sometimes, a barrier to receiving HIV care over telephone. Most respondents also had access to the privacy necessary for video visits (87%, *n* = 236). Of the 35 people who did not always have access, 38% (*n* = 13) reported that this lack of consistent privacy was always, and 29% (*n* = 10) reported it was sometimes, a barrier to receiving HIV care over video.

### Technological literacy

The median technological literacy rating of the population sampled was 10 (highest level of technological literacy) across all devices. Most individuals (69%, *n* = 188) had a technological literacy rating of at least 7 across all prompts for all devices. Only 3% (*n* = 8) of PLHIV gave themselves a 5 or less across all prompts for a device that they owned.

Approximately three-quarters of the participants (77%, *n* = 205) used their telephone for personal video calls, while 61% (*n* = 97) did so on a tablet, laptop, or desktop computer. While both clinics offer an online portal for health and medical information, one-third of all participants were not aware this portal existed (31%, *n* = 83).

### Attitudes towards and experiences with telemedicine

**Fig. 1:**
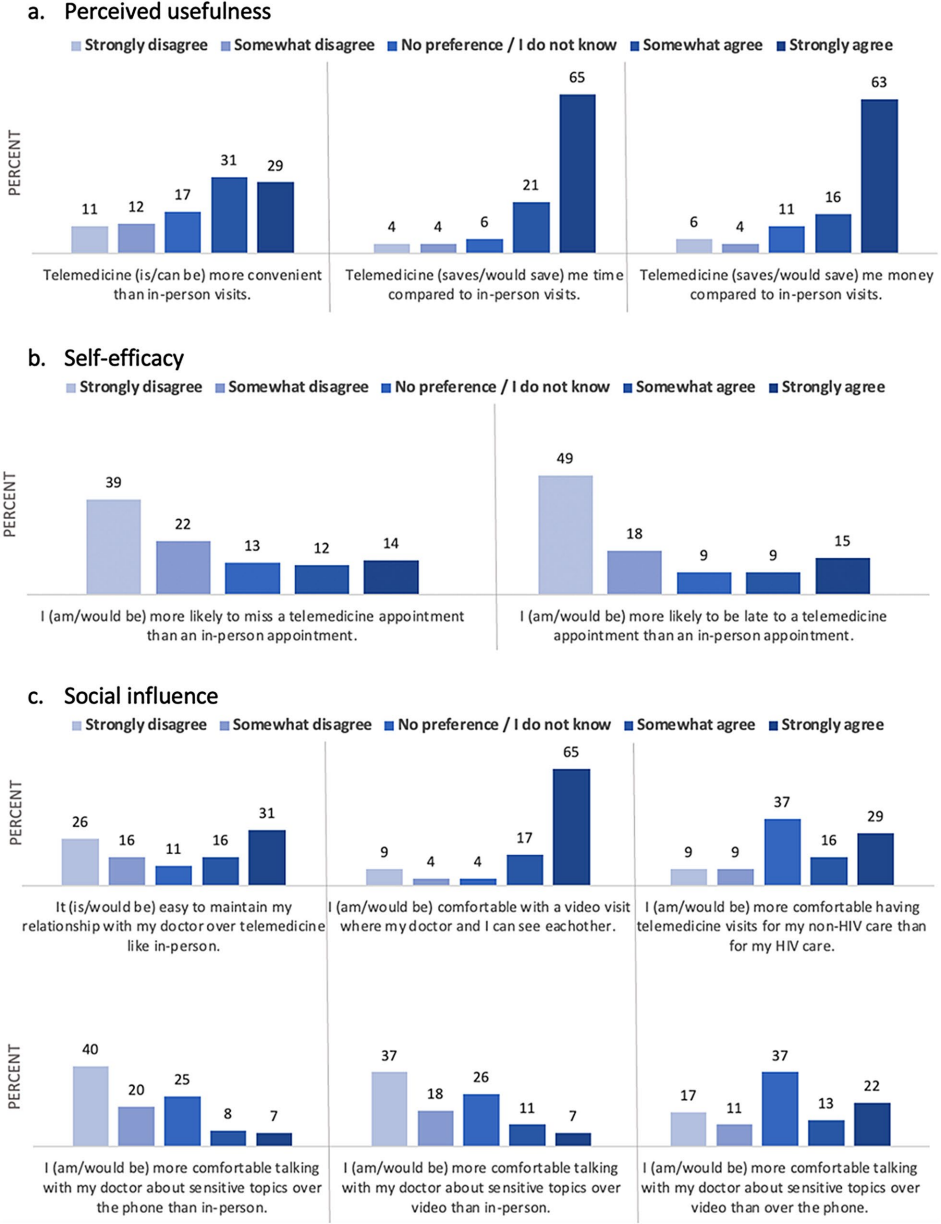
Attitudes towards and experiences with telemedicine (*N* = 271)

Sixty percent of PLHIV (*n* = 162) had used telemedicine at the time of the survey and 40% (*n* = 109) had not and therefore answered questions about telemedicine from a hypothetical perspective. Respondents largely felt that telemedicine saved (or would save, hypothetically) them time (86%, *n* = 232) and money (79%, *n* = 215) compared to an in-person visit (Fig. 1a). When participants were asked if they were (or would be, hypothetically) more likely to miss a telemedicine appointment compared to an in-person appointment, most (61%, *n* = 165) disagreed (Fig. 1b). They also felt that they were not (or would not be) more likely to be late to a telemedicine appointment compared to in person (67%, *n* = 181).

Most participants (70%, *n* = 164) felt that telemedicine was (or would be, hypothetically) more convenient than in-person visits (Fig. 1a). Among those for whom their preferred language was Spanish as well as those with grade school as the highest education completed, in-person visits were also more commonly reported as convenient (57% of Spanish-speaking PLHIV versus only 18% of English-speaking PLHIV, *p* < *0.001;* 51% of grade school versus 16% of high school graduates, *p* < *0.001*, respectively). Among those older than 45 years, in-person visits were more commonly reported as convenient (28% of participants > 45 versus 17% of participants ≤ 45, *p* = *0.001).* Gender and housing status were not associated with perceptions of convenience.

Most PLHIV (82%, *n* = 222) stated that they would feel comfortable having their doctor be able to see them on a video call (Fig. 1c). Identifying as a woman (cisgender or transgender) and grade school as the highest level of education were associated with lower levels of comfort with video visits (27% of women versus 10% of men, *p* < *0.001;* 25% of grade school versus 11% of high school graduates, *p* < *0.001*,* respectively*). Age, preferred language, and housing status were not associated with comfort level around video visits.

Responses were varied regarding the ability to maintain their relationship with their clinician over telemedicine. Approximately half of participants (47%, *n* = 128) reported it was or would be easy to maintain a relationship, but 41% (*n* = 112) disagreed with this statement. Most PLHIV preferred to discuss sensitive topics (i.e., substance use, relationship issues, etc.) in person rather than telephone (60%, *n* = 164) or in person rather than a video visit (55%, *n* = 151), while about one-quarter (*n* = 67) had no preference. In addition, when asked about comfort level of telemedicine for HIV care versus non-HIV care, 45% (*n* = 121) felt more comfortable with telemedicine for non-HIV care and 37% (*n* = 100) stated no preference (Fig. 1c).

PLHIV were asked what their preferred mix of HIV care appointments would be for the future. Most (71%, *n* = 193) reported wanting some in-person visits mixed with some telemedicine visits, while about a quarter of respondents only wanted in-person visits (24%, *n* = 65) (Table 4). Of those who wanted at least some telemedicine for their HIV care, approximately two-thirds (65%, *n* = 134) wanted a mix of telephone and video visits while 17% (*n* = 34) wanted only telephone and 15% (*n* = 31) only video. Identifying as a woman and age > 45 were associated with a preference for all in-person visits (38% of women versus 21% of men, *p* = 0.041; 27% of participants aged > 45 versus 9% aged ≤ 45, *p* = 0.043). Grade school as the highest level of education was also associated with a preference for all in-person visits (40% of grade school versus 20% of high school graduates, *p* = 0.001). Preferred language and housing status were not associated with a preference for in-person visits.

**Table 4:** PLHIV preferences for telemedicine for HIV care (*N* = 271)

	Overall	Clinic 1	Clinic 2
	(*N* = 271)	(*N* = 136)	(*N* = 135)
**Preferred mix of HIV care appointments in future**, *N* (%)			
All in person	65 (24)	29 (21)	36 (27)
All telemedicine	9 (3)	5 (4)	4 (3)
Some in person and some telemedicine	193 (71)	99 (73)	94 (69)
No preference/I do not know	4 (1)	3 (2)	1 (1)
**Preferred types of telemedicine visits**, * *N* (%)			
All telephone/audio	34 (17)	13 (12)	21 (21)
All video	31 (15)	15 (14)	16 (16)
Some telephone/audio and some video	134 (65)	77 (72)	57 (58)
No preference/I do not know	7 (3)	2 (2)	5 (5)

*N = 206

When these same analyses were limited to only those with actual experience using telemedicine (phone or video), the results were similar except that grade school as the highest level of education was no longer statistically associated with lower levels of comfort with video visits, although the trend was in the same direction (22% of grade school versus 9% of high school graduates, *p* = *0.189).*

## Discussion

We found that PLHIV cared for in two FQHCs in Los Angeles showed high interest in and satisfaction with telemedicine for HIV care, which was delivered largely in the form of telephone visits during a mature phase of the COVID-19 pandemic. Similar data have been reported from private and academic settings in which high satisfaction has been demonstrated with telemedicine for primary and specialty care, although these settings utilized video telemedicine predominantly [19, 20]. Three-quarters of participants surveyed in our population were interested in having the option of telemedicine in addition to in-person visits in the future – even with COVID-19 no longer posing barriers to in-person care – and most respondents had a preference for receiving telemedicine as a mixture of telephone and video visits.

Our experience showing PLHIV receive their telemedicine visits predominantly via telephone (93%) is similar to other literature from FQHCs in California and across the U.S. during the pandemic [3, 10–12]. There are several reasons why video use may have been low in our population despite theoretical interest by those surveyed. Our previous qualitative work with clinicians and staff at these same clinics revealed lack of an integrated video telemedicine platform in the electronic medical record, which made video visits burdensome and, at times, impossible, due to technical issues; additionally, many clinicians believed telephone was easier for clients and therefore did not routinely offer video [10]. Ganguli et al. evaluated telemedicine preferences among Medicare beneficiaries and found that, of 1,057 patients offered telemedicine, 68% accepted. Of those who accepted, 43% selected telephone and 25% video, with the remaining 32% selecting a mix of both modalities. The authors conclude that an “often overlooked idea” is that patients may prefer telephone over video visits [21]. In a 2021 nationally representative survey, most respondents (66.5%) said they were interested in at least some future video care for primary health care, but when given the choice between in person or video for a non-emergency visit, more than half (53%) chose in person, with individuals selecting video more likely to be younger (< 39 years) and have a higher income [22]. Our study similarly found that younger adults (≤ 45 years) were more likely to select at least some telemedicine for their future care, while older adults were more likely to prefer all in-person visits.

Participants in our sample answered questions about video telemedicine from a largely hypothetical perspective given low video use at the time our survey was performed. Despite theoretical interest in video, PLHIV may make different choices when faced with an actual decision to use video (versus telephone or in person) for reasons such as simplicity and/or privacy. Further research is needed from FQHCs to understand uptake of video telemedicine in the context of routine offer of this modality.

Our study participants liked telemedicine for its convenience and cost savings, and most reported that it was easier to be on-time for telemedicine appointments compared to in person. Similar findings have been reported in the literature, with data from an FQHC in Texas showing a reduction in missed appointments with telemedicine [23]. Nationally representative data from the first year of the pandemic also found that most adults (18 to 64 years) with public insurance did not have out-of-pocket costs for their telemedicine visits (81.5%), thought it was easy to schedule telemedicine visits at a convenient time (83.6%), and experienced a shorter wait time compared to in person (67.7%) [23, 24].

Lack of technological resources and skills have been raised as barriers to the use of telemedicine in FQHCs [3, 10, 13]. However, in our study, almost all PLHIV had a smartphone (95%) with reliable cellular data/Wi-Fi, self-rated technological literacy was high, and most participants reported using their devices for personal video calls. Most (66%) also either owned a tablet, laptop, or desktop computer or would always be able to borrow one from a friend/family member for video telemedicine. This device coverage is similar to that found among patients cared for in the largest safety net clinic in Northern California, in which 90.3% owned a smartphone and 45.7% owned a computer [25]. Despite high levels of technological literacy in our study, people may still face barriers to engagement in video telemedicine, particularly if they are required to download applications or use unfamiliar software. Studies suggest that familiarizing patients with the video visit technology may positively impact their experience and interest in engaging in this type of care [26–28]. For FQHCs and similar clinical contexts that have faced challenges with the implementation of video telemedicine, working with clinicians and staff to consistently offer PLHIV the opportunity to practice a video visit could help ensure equitable access, increase uptake, and improve completion rates for this type of care. Additionally, access to technology may vary over weeks or months based on individual circumstances; therefore, each offer of telemedicine should be coupled with an understanding of the person’s capacity to participate at that point in time.

We found that sociodemographic factors may serve as barriers to participation in telemedicine, including privacy and lack of English literacy. Privacy was raised as a challenge most commonly by participants with housing instability (15% of our study population was housing unstable). Qualitative data have shown that living in shared housing can be a significant barrier to the use of telemedicine, particularly for individuals who have not disclosed to members of the household [10]. Our data are consistent with a study at an FQHC in Houston in which lack of privacy at home limited PLHIV’s telemedicine utilization [26]. While our sample size of Spanish-speaking participants was small, we found Spanish-speakers more commonly described in-person visits as more convenient than telemedicine.

Lack of English-language literacy as a barrier to telemedicine has been shown in other studies [13, 21, 28]. Several studies and commentaries have suggested ways to address the structural barriers to telemedicine for people who lack English literacy, including use of trained interpreters during telemedicine visits, simple video platforms that can be easily accessed by individuals with low English literacy, and technological literacy/telemedicine training in multiple languages [29–31]. Certain HIPAA-compliant applications, including Doximity [32] and Zoom [33], allow for an additional participant, so an interpreter could join in real-time. While this may require an additional step in the workflow for clinicians and clinic staff, use of this strategy would expand the availability of telemedicine services to those who face language barriers and would advance equitable access in clinical contexts such as FQHCs, where health disparities tend to be the greatest.

Respondents in our study reported several limitations to telemedicine, particularly surrounding sensitive conversations (e.g., around sexually transmitted infections, substance use disorders, and relationship issues, among others), which they generally favored to have in person. Individuals who share living spaces with roommates, friends, or family, or who experience intimate partner violence can face additional barriers to having sensitive conversations via telemedicine. In these instances, telemedicine could result in inadvertent disclosure of sensitive health information and could be a risk for individuals’ safety. Therefore, ensuring clients are given choices about how they receive care, particulary when sensitive conversations are anticipated, is critical for protecting privacy.

Individuals had diverse perspectives around how telemedicine influenced their relationships with clinicians, with interviewees either feeling strongly that telemedicine was harmful or beneficial to the relationship. PLHIV’s relationships with their care team are of the utmost importance for any successful health service delivery approach. Our data underscore the necessity of a person-centered approach, as there is no “one-size-fits-all” for how telemedicine is incorporated into HIV care [10, 34].

### Limitations

We surveyed PLHIV engaged in care who agreed to participate in a survey study, and therefore our findings about telemedicine cannot be generalized to all individuals receiving care or those who are disengaged due to significant challenges to care access. We had a small number of Spanish-speaking participants in our sample and therefore had limited power to detect differences in telemedicine attitudes/experiences between English and Spanish-speaking individuals. Participants were asked to self-report technological literacy, which may skew responses towards reporting higher levels due to social desirability bias [35]. At the time of our survey, both study sites were predominantly using telephone telemedicine and only 60% of respondents had ever used any form of telemedicine; therefore, a large number of responses were hypothetical rather than informed by practical experience. We did use a sensitivity analysis to explore correlates of actual use, and found overall very similar findings to the full sample that included both actual use and hypothetical preferences. We were unable to evaluate whether there was an association between duration on ART and attitudes about and experiences with telemedicine for HIV visits given most participants had been on ART for many years. Future research should include individuals more recently diagnosed with HIV. Lastly, we are not able to evaluate how use of telemedicine may influence clinical outcomes such as engagement, retention, and viral load suppression over time. Understanding the impact that telemedicine (both telephone and video) has on clinical outcomes among PLHIV cared for in FQHCs will be important for consideration for whether to (and how to) scale up this form of care.

## Conclusions

PLHIV in our study showed high satisfaction with telemedicine, which was delivered as predominantly telephone visits in two FQHCs in Los Angeles during a mature phase of the COVID-19 pandemic. Respondents expressed high interest in using telemedicine for future HIV care, including video visits. While individuals raised concerns about certain challenges with telemedicine, such as feeling more comfortable with discussing sensitive topics in person, they found that telemedicine made it easier to make their appointments on-time and saved them time and money. Future research is needed on the barriers to and potential benefits of video visits, as compared to telephone visits, for telemedicine HIV care in FQHCs. Additional studies are needed to determine telemedicine’s impact on HIV outcomes, such as engagement in care and viral suppression.

## Electronic Supplementary Material

Below is the link to the electronic supplementary material.

Supplementary Material 1

## Data Availability

Data and materials used for this study are available upon request and approval by the corresponding author and study PI.

## Abbreviations

ART: Antiretroviral therapy
COVID 19: Coronavirus disease 2019
CMS: Centers for Medicare & Medicaid Services
FQHC: Federally qualified health center
GED: General Educational Development Test
PLHIV: People living with HIV
SPA: Service Planning Area
