# Supplementary material for: Telemedicine for HIV care: a cross-sectional survey of people living with HIV receiving care at two federally qualified health centers in Los Angeles during a mature phase of the COVID-19 pandemic
Source: BMC Infect Dis. 2024 Dec 31;24:1481. doi: 10.1186/s12879-024-10351-x (PMC11687067; doi:10.1186/s12879-024-10351-x)
Supplement: Supplementary file 1 — Supplementary Material 1 [file 12879_2024_10351_MOESM1_ESM.docx]

*Supplementary Table 1. Patient Baseline Survey*

| SOCIO-DEMOGRAPHIC | |
| --- | --- |
| 1. What is your date of birth? | MM/DD/YYYY |
| 1. What gender do you identify as? | - Female - Male - Transgender female - Transgender male - Gender nonconforming - Other (please describe): __________ - Prefer not to answer |
| 1. What is your race/ethnicity? Select all that apply. | - Black/African-American - Hispanic or Latino/a - Asian - Native American - White/Caucasian - Other (please describe): __________ - Prefer not to answer |
| 1. What language do you prefer to use when communicating with your clinician? 2. On a scale of 1-5, how comfortable are you with reading in this language? | - English - Spanish - Other: __________   1 2 3 4 5  *Not Totally at all comfortable comfortable* |
| 1. If English is not your preferred language, on a scale of 1-5, how comfortable are you with speaking and understanding English? 2. On a scale of 1-5, how comfortable are you with reading English? | 1 2 3 4 5  *Not Totally at all comfortable comfortable*  1 2 3 4 5  *Not Totally at all comfortable comfortable* |
| 1. What sexual orientation do you most identify with? | - Heterosexual/straight - Gay - Lesbian - Queer - Bisexual - Pansexual - Asexual - Other: __________ - Prefer not to answer |
| 1. What is the highest level of school you have completed? | - None - Some school but did not complete high school/GED - High school/GED - College/university - Graduate studies - Do not know - Prefer not to answer |
| 1. What is your employment status? | - Working part-time - Working full-time - Retired - On disability - Not working - Prefer not to answer |
| 1. Where have you regularly slept in the past three months (pick the option that best represents the past three months)? | - Housing you own - Housing you rent - Housing you share with friends, family or acquaintances but do not pay for - Shelter, safe haven, or transitional housing - Car, in a tent, on the street, or under a bridge - Other: __________ - Prefer not to answer |
| 1. If currently in stable housing over the past three months [skip if living in transitional or unhoused environment], 2. What is the number of individuals in your household currently who are less than 18 years old? 3. What is the number of individuals in your household currently who are 18 years or older? | ###   - Do not know - Prefer not to answer   ###   - Do not know - Prefer not to answer |
| 1. Are you worried that in the next three months you may not have stable housing that you own, rent, or stay in as part of a household? | - Yes, very worried - Somewhat worried - No, not worried at all - Prefer not to answer |
| 1. Do you have access to privacy for phone/audio calls about your HIV care in your current housing space? 2. If sometimes or no, have you found this lack of consistent privacy a barrier to having your HIV care over the phone/audio? | - Yes, always - Sometimes - No, never - Prefer not to answer - Yes, always - Sometimes - No, never - N/A – I have not had any HIV care over the phone/audio - Prefer not to answer |
| 1. Do you have access to privacy for video calls about your HIV care in your current housing space? 2. If sometimes or no, have you found this lack of consistent privacy a barrier to having your HIV care over video? | - Yes, always - Sometimes - No, never - Prefer not to answer - Yes, always - Sometimes - No, never - N/A – I have not had HIV care over video calls - Prefer not to answer |

| TELEMEDICINE | |
| --- | --- |
| 1. Do you own a phone that currently can make and receive calls? 2. If yes, what kind of phone do you have? 3. Do you use video calls on your phone to speak with family or friends, for work, or for any other purpose? | - Yes - No - Do not know - Prefer not to answer - Landline - Cell phone - Smartphone - Other: __________ - Do not know - Prefer not to answer - Yes - No - N/A, I only have a landline or cell phone that isn’t capable of video calls - Prefer not to answer |
| 1. Has this or any other phone been available to you consistently over the past 3 months? | - Yes - No - Prefer not to answer |
| 1. Do you currently own a tablet or a laptop/computer? 2. If yes, do you use your tablet or laptop/computer for video calls with family of friends, for work, or for any other purpose? 3. If no, can you borrow a tablet or laptop/computer (from a friend or family member) for an HIV telemedicine visit? | - Yes - No - Prefer not to answer - Yes - No - Prefer not to answer - Yes, consistent access (always) - Yes, inconsistent access (sometimes) - No access (never) - Prefer not to answer |
| 1. Do you currently have access to a reliable Wi-Fi or mobile data connection to use for telemedicine visits? | - Yes, always - Yes, sometimes - No, never - Prefer not to answer |
| 1. Have you ever had any telephone/audio or video telemedicine visits for your HIV care? This is defined as any HIV care visit where you spoke to your HIV primary care clinician by telephone/audio or by video instead of coming into clinic. 2. On a scale from 1 to 4, with 1 being Strongly disagree and 4 being Strongly agree, how do you feel about the following statements? (Note: if they have not had experience, these should still be asked based on the participant’s thoughts/feelings) 3. Telemedicine for HIV care is (or can be) more convenient than in-person visits. 4. I am (or would be) more likely to miss an HIV care telemedicine appointment than an in-person appointment. 5. I am (or would be) more likely to be late to an HIV care telemedicine appointment than an in-person appointment. 6. Telemedicine for my HIV care saves me (or would save me) time compared to in-person visits. 7. Telemedicine for my HIV care saves me (or would save me) money compared to in-person visits.   Note: participants should consider the total money for their visit, such as the money they spend on traveling to the clinic or the money they lose from taking time off from work.   1. Thinking back to when I was first diagnosed with HIV, I would have been okay with having my first HIV care visits over telemedicine instead of in-person. 2. It is (or would be) more difficult to maintain the kind of relationship I like best with my doctor during telemedicine HIV care versus in-person. 3. I feel (or would feel) comfortable being on a video telemedicine call for HIV care with my doctor where they can see me and I can see them. 4. I feel (or would feel) more comfortable talking with my HIV care doctor about sensitive topics over a phone/audio call than in-person. 5. I feel (or would feel) more comfortable talking with my HIV care doctor about sensitive topics over a video call than in-person. 6. I feel (or would feel) more comfortable talking with my HIV care doctor about sensitive topics over video call than phone/audio call.   [NEED SKIP PATTERN HERE IF HAS NEVER RECEIVED TELEMED, Q41and/or Q42 ARE SKIPPED]   1. I feel satisfied with the quality of care I’ve received on my HIV phone/audio visits. 2. I feel satisfied with the quality of care I’ve received on my HIV video visits. | - Yes, telephone/audio only - Yes, video only - Yes, both telephone/audio and video - No - Prefer not to answer - 1 – Strongly disagree - 2 – Disagree - 3 – Agree - 4 – Strongly agree - No preference / I do not know - 1 – Strongly disagree - 2 – Disagree - 3 – Agree - 4 – Strongly agree - No preference / I do not know - 1 – Strongly disagree - 2 – Disagree - 3 – Agree - 4 – Strongly agree - No preference / I do not know - 1 – Strongly disagree - 2 – Disagree - 3 – Agree - 4 – Strongly agree - No preference / I do not know - 1 – Strongly disagree - 2 – Disagree - 3 – Agree - 4 – Strongly agree - No preference / I do not know - 1 – Strongly disagree - 2 – Disagree - 3 – Agree - 4 – Strongly agree - No preference / I do not know - 1 – Strongly disagree - 2 – Disagree - 3 – Agree - 4 – Strongly agree - No preference / I do not know - 1 – Strongly disagree - 2 – Disagree - 3 – Agree - 4 – Strongly agree - No preference / I do not know - 1 – Strongly disagree - 2 – Disagree - 3 – Agree - 4 – Strongly agree - No preference / I do not know - 1 – Strongly disagree - 2 – Disagree - 3 – Agree - 4 – Strongly agree - No preference / I do not know - 1 – Strongly disagree - 2 – Disagree - 3 – Agree - 4 – Strongly agree - No preference / I do not know - 1 – Strongly disagree - 2 – Disagree - 3 – Agree - 4 – Strongly agree - No preference / I do not know - N/A – I have not received phone/audio calls (only video) - 1 – Strongly disagree - 2 – Disagree - 3 – Agree - 4 – Strongly agree - No preference / I do not know - N/A – I have not received video calls (only phone/audio) |
| 1. In the future, what is your preferred mix of HIV care appointment types? | - All in-person - All telemedicine - Some in-person and some telemedicine - Not sure - No preference |
| 1. How would you like to receive your telemedicine care? | - All telephone/audio - All video - Some telephone/audio and some video - Not sure - No preference - I do not want to have telemedicine appointments |
| 1. In the last year, have you had telemedicine visits for any other kind of non-HIV health care? 2. If yes, did you receive this non-HIV care via telephone/audio call or video call? | - No - Yes: - Mental health care - Case management - Acute care (sinus infection, cold, flu, etc.) - COVID-related care - Specialty care (cardiology, gynecology, nephrology, etc) - Other: __________ - Do not know - Prefer not to answer - Telephone/audio only - Video only - Both - Do not know - Prefer not to answer |
| 1. On a scale from 1 to 4, with 1 being Strongly disagree and 4 being Strongly agree, how do you feel about the following statement? 2. I feel (or would feel) more comfortable having telemedicine visits for my non-HIV care than for my HIV care. | - 1 – Strongly disagree - 2 – Disagree - 3 – Agree - 4 – Strongly agree - No preference / I do not know |
| 1. On a scale from 1 to 10, with 1 being Not at all able to and 10 being Totally able to, how would you rate your abilities to perform the following actions on your own? 2. I can increase or decrease the brightness on my phone. 3. I can increase or decrease the volume on my phone. 4. I can download a new app on my phone. 5. I can text from my phone. 6. I can email from my phone. 7. I can answer a video call on my phone. 8. I can use my phone for a video call visit with my doctor. 9. I can increase or decrease the brightness on my tablet or laptop/computer. 10. I can increase or decrease the volume on my tablet or laptop/computer. 11. I can download a new app or computer program to my tablet or laptop/computer. 12. I can email from my tablet or laptop/computer. 13. I can set up and use my tablet or laptop/computer for a video call visit with my doctor. | 1 2 3 4 5 6 7 8 9 10  *Not Totally at all able able to*  *to*   - Not applicable - Prefer not to answer   1 2 3 4 5 6 7 8 9 10  *Not Totally at all able able to*  *to*   - Not applicable - Prefer not to answer   1 2 3 4 5 6 7 8 9 10  *Not Totally at all able able to*  *to*   - Not applicable - Prefer not to answer   1 2 3 4 5 6 7 8 9 10  *Not Totally at all able able to*  *to*   - Not applicable - Prefer not to answer   1 2 3 4 5 6 7 8 9 10  *Not Totally at all able able to*  *to*   - Not applicable - Prefer not to answer   1 2 3 4 5 6 7 8 9 10  *Not Totally at all able able to*  *to*   - Not applicable - Prefer not to answer   1 2 3 4 5 6 7 8 9 10  *Not Totally at all able able to*  *to*   - Not applicable - Prefer not to answer   1 2 3 4 5 6 7 8 9 10  *Not Totally at all able able to*  *to*   - Not applicable - Prefer not to answer   1 2 3 4 5 6 7 8 9 10  *Not Totally at all able able to*  *to*   - Not applicable - Prefer not to answer   1 2 3 4 5 6 7 8 9 10  *Not Totally at all able able to*  *to*   - Not applicable - Prefer not to answer   1 2 3 4 5 6 7 8 9 10  *Not Totally at all able able to*  *to*   - Not applicable - Prefer not to answer   1 2 3 4 5 6 7 8 9 10  *Not Totally at all able able to*  *to*   - Not applicable - Prefer not to answer |
| 1. Do you have access to an online patient portal from your clinic where you receive HIV care? If no, skip to 68. 2. If yes, on a scale from 1 to 10, with 1 being Not at all able to and 10 being Totally able to, how would you rate your abilities to perform the following actions on your own? 3. I can access my patient portal on my phone. 4. I can access my patient portal on my tablet or laptop/computer. 5. I can use my patient portal to see my lab results. 6. I can use my patient portal to send a message to my doctor. | - Yes - No - Do not know   1 2 3 4 5 6 7 8 9 10  *Not Totally at all able able to*  *to*   - Not applicable - Prefer not to answer   1 2 3 4 5 6 7 8 9 10  *Not Totally at all able able to*  *to*   - Not applicable - Prefer not to answer   1 2 3 4 5 6 7 8 9 10  *Not Totally at all able able to*  *to*   - Not applicable - Prefer not to answer   1 2 3 4 5 6 7 8 9 10  *Not Totally at all able able to*  *to*   - Not applicable - Prefer not to answer |

| MEDICAL HISTORY | |
| --- | --- |
| 1. What was the year of your HIV diagnosis?   [okay to approximate if not certain] | - YYYY - Do not know - Prefer not to answer |
| 1. When did you first start antiretroviral therapy (ART)? | - MM/YYYY - Do not know - Prefer not to answer |

| CLINIC COSTS | |
| --- | --- |
| 1. What is the most common mode of transportation you use to get to the clinic? 2. How much time do you spend traveling one-way to the clinic on your primary mode of transportation? Round to the nearest 15-minutes. Think of the most recent few times you traveled to the clinic when you answer this question to come up with your estimate. | - Public transportation - Personal vehicle that I drive myself, like a car or motorcycle - Personal vehicle that a friend/family member drives - Ridesharing apps, like Uber or Lyft - Walking - Bicycle - Other (i.e., Access rides, ambulette services, split between modes): __________ - Do not know - Prefer not to answer   HH:MM   - Do not know - Prefer not to answer |
| 1. Do you pay anything for transportation to and from the clinic (based on the most common type of transport above)? | - Yes - No - Do not know - Prefer not to answer |
| 1. If yes, how much do you spend one-way to the clinic on your primary mode of transportation per visit? Round to the nearest dollar and think about the last time you used this most common type of transport. | $$$   - Do not know - Prefer not to answer |
| 1. Do you usually take time off from work in order to attend your HIV appointments? | - Yes - No - Sometimes - Prefer not to answer |
| 1. If yes, is this time paid or unpaid? 2. If unpaid or it varies, what is the average lost wage for the time you take off from work for an HIV appointment? Round to the nearest dollar. Think of the most recent few times you missed work when you answer this question to come up with your estimate. | - Paid - Unpaid - It varies - Do not know - Prefer not to answer   $$$   - Do not know - Prefer not to answer |
| 1. Are there any additional costs or things you spend money on to be able to attend your HIV visits at the clinic, such as childcare or an appointment companion? 2. If yes, what is the thing you spend money on? 3. What is the average additional cost for this/these for one HIV appointment (round to nearest dollar)? | - Yes - No - Do not know - Prefer not to answer - Childcare costs - Appointment companion - Other: __________ - Do not know - Prefer not to answer   $$$   - Do not know - Prefer not to answer |
| 1. Thinking about the last few times you went to the clinic in-person, how much time did you spend there, from the moment you walked in the clinic door to the moment you walked out? Round to the nearest half hour. | HH:MM   - Do not know - Prefer not to answer |
| 1. How much do you pay for your phone each month, on average? This should include the cost of the actual phone if on a payment plan plus the cost of data together. 2. If $$$ > $0, how much of what you pay is just for your data plan? 3. Calculate how much of what the participant pays is just for their phone payment = Q81-Q82. 4. How do you pay for your mobile data? 5. On a scale from 1 to 10, with 1 being Not at all difficult and 10 being Extremely difficult, how difficult is it to financially maintain your phone? 6. On a scale from 1 to 10, with 1 being Not at all difficult and 10 being Extremely difficult, how difficult is it to financially maintain your mobile data? | $$$   - Do not know - Prefer not to answer   $$$   - Do not know - Prefer not to answer   $$$   - Do not know - Prefer not to answer - Monthly - Pay as you go - Other: __________ - Do not know - Prefer not to answer   1 2 3 4 5 6 7 8 9 10 *Not Totally*  *at all able able to to*   - Not applicable - Prefer not to answer   1 2 3 4 5 6 7 8 9 10 *Not Totally*  *at all able able to to*   - Not applicable - Prefer not to answer |
| 1. Do you pay for WiFi? 2. If yes or sometimes, how do you pay for your WiFi? 3. How much do you pay for your WiFi each month, on average? Round to the nearest dollar and think about the last few months to come up with your estimate. 4. On a scale from 1 to 10, with 1 being Not at all difficult and 10 being Extremely difficult, how difficult is it to financially maintain your WiFi? | - Yes - Sometimes - No - Do not know - Prefer not to answer - Monthly - Pay as you go - Other: __________ - Do not know - Prefer not to answer   $$$   - Do not know - Prefer not to answer   1 2 3 4 5 6 7 8 9 10 *Not Totally*  *at all able able to to*   - Not applicable - Prefer not to answer |
